# Association between vascular FDG uptake during follow-up and the development of thoracic aortic aneurysms in giant cell arteritis

**DOI:** 10.3389/fmed.2024.1384533

**Published:** 2024-03-20

**Authors:** Daniel Blockmans, Lien Moreel, Albrecht Betrains, Steven Vanderschueren, Walter Coudyzer, Lennert Boeckxstaens, Koen Van Laere

**Affiliations:** ^1^Department of General Internal Medicine, University Hospitals Leuven, Leuven, Belgium; ^2^Department of Microbiology, Immunology and Transplantation, KU Leuven, Leuven, Belgium; ^3^European Reference Network for Immunodeficiency, Autoinflammatory, Autoimmune and Pediatric Rheumatic Disease (ERN-RITA), Utrecht, Netherlands; ^4^Department of Radiology, University Hospitals Leuven, Leuven, Belgium; ^5^Division of Nuclear Medicine, University Hospitals Leuven, Leuven, Belgium; ^6^Department of Imaging and Pathology, Nuclear Medicine and Molecular Imaging, KU Leuven, Leuven, Belgium

**Keywords:** giant cell arteritis (GCA), PET, aorta, aneurysm, vasculitis

## Abstract

**Background:**

A positive PET scan at diagnosis was associated with a greater yearly increase in ascending and descending aortic diameter and thoracic aortic volume in patients with giant cell arteritis (GCA). Radiologic and histopathologic vascular abnormalities persist in a subset of treated patients despite clinical remission. The aim of this study was to evaluate the association between vascular FDG uptake during follow-up and the development of thoracic aortic aneurysms.

**Methods:**

We recently performed a prospective cohort study of 106 GCA patients, who underwent FDG PET and CT imaging at diagnosis and CT imaging yearly for a maximum of 10 years. In this *post hoc* analysis, GCA patients who also have had FDG PET imaging during follow-up were included. PET scans were visually scored (0–3) at 7 vascular areas. PET scans were considered positive in case of FDG uptake ≥grade 2 in any large vessel.

**Results:**

Eighty-eight repeat PET scans were performed in 52 out of 106 GCA patients, who were included in the original prospective cohort. Fifty-five (63%) PET scans were done at the time of a relapse and 33 (38%) were done while in remission. Nine out of ten patients with an incident thoracic aortic aneurysm had both a positive PET scan at diagnosis and during follow-up.

**Conclusion:**

In addition to the intensity and extent of the initial vascular inflammation, ongoing aortic inflammation may contribute to the development of thoracic aortic aneurysms in GCA. However, this hypothesis should be confirmed in a large prospective trial with repeat PET scans at predefined time points during follow-up.

## Introduction

Recently, we reported our results of a prospective study investigating the association between vascular ^18^F-fluorodeoxyglucose (FDG) uptake at diagnosis and changes in aortic dimensions in 106 giant cell arteritis (GCA) patients (1). We found that GCA patients with a positive FDG positron emission tomography (PET) scan at diagnosis (defined as a scan with FDG uptake grade 2 or higher in any large vessel) had a greater increase in ascending and descending aortic diameter and thoracic aortic volume compared to those with a negative scan. There were no differences in abdominal aortic dimensions. In addition, higher total vascular score (defined as the sum of vascular scoring 0–3 in 7 vascular areas) was associated with a greater yearly increase in thoracic aortic diameters and volume. During follow-up, 15 patients developed a new thoracic aortic aneurysm, most frequently in the descending aorta. Fourteen of these 15 thoracic aortic aneurysms were seen in patients who were PET positive at diagnosis and 87% of the patients had thoracic aortic dilatation in a region with elevated FDG uptake at diagnosis. There was no association between the development of a thoracic aortic aneurysm and the duration of glucocorticoid treatment, the cumulative glucocorticoid dose during the first two years after diagnosis, the use of glucocorticoid-sparing agents or the relapse rate. Hence, we concluded that the intensity and extent of the initial inflammation determines the risk for subsequent aortic dilatation.

However, initial aortic wall inflammation may not be the only determining factor in aneurysm formation. Radiologic vascular abnormalities persist in a subset of treated GCA patients despite clinical remission (2–5). It is unclear whether this represents active inflammation or vascular remodeling. In a recent study, histopathological evaluation of aortic surgical specimens after aortic repair showed active aortitis in most GCA patients, who were treated and were in clinical remission (6). This finding suggests that steroid-resistant, smoldering aortic inflammation possibly contributes to the development of thoracic aortic aneurysms in GCA.

In order to test this hypothesis, we wanted to evaluate if there was an association between persistent vascular FDG uptake and the development of thoracic aortic aneurysms as an exploratory *post hoc* analysis of the previously published prospective cohort of GCA patients (1).

## Methods

This study was a *post hoc* analysis of the observational prospective study investigating the association between vascular FDG uptake at diagnosis and change in aortic dimensions in 106 GCA patients. Details on the study protocol and outcomes have been recently published (1). Briefly, we included GCA patients, who were evaluated in the department of General Internal Medicine of the University Hospitals Leuven between 2012 and 2020 and who had undergone FDG PET imaging at diagnosis within 3 days after the initiation of glucocorticoids. Patients with a previous diagnosis of GCA, in whom a PET scan and yearly computed tomography (CT) of the aorta were available, were also included. In this *post hoc* analysis, only patients who also had undergone FDG PET imaging during follow-up, were included. PET scans during follow-up were not part of the initial protocol, but were done at the discretion of the treating physician as judged necessary in real life, for instance when the treating physician was in doubt whether there was a relapse or not.

Patients underwent a CT scan of the thorax and abdomen at diagnosis and yearly thereafter for a maximum of 10 years. The aortic diameter was measured perpendicular to the axis of blood flow at 6 different levels (ascending aorta, aortic arch, descending thoracic aorta, suprarenal, juxtarenal and infrarenal aorta) and the thoracic and abdominal aortic volumes were measured. The ascending aorta was considered aneurysmatic when the diameter was ≥45 mm, the aortic arch ≥40 mm and the descending aorta ≥35 mm (7, 8).

The study was conducted in accordance with the Declaration of Helsinki and approved by the ethical committee of the University Hospitals Leuven.

### PET imaging and analysis

Patients were required to fast for at least 6 h before intravenous injection of 4–5 MBq/kg of ^18^F-FDG, and glycemia levels were determined in all patients (as per procedure, should be <140 mg/dl). A whole-body PET scan was performed 60 min after tracer administration. PET scans were performed between 2003 and 2020, consecutively acquired on four different PET cameras (ECAT HR + PET, Hirez Biograph 16 PET/CT, Truepoint Biograph 40 PET/CT [Siemens, Knoxville, TN, USA] or Discovery MI-4 PET/CT [GE, Milwaukee, WI, USA]). Since gamma rays from the positron annihilation in PET are absorbed by the body, a correction for this attenuation allows quantitatively accurate judgment of internal regions in the body. On the PET/CT systems, either a low-dose CT scan or a diagnostic, high-dose CT scan was performed immediately before PET acquisition. The CT scan was used for attenuation correction and for anatomical localization. For the older HR + acquisitions, no attenuation correction was performed. Attenuation-corrected PET images were thus only available for the scans performed on a PET/CT system (*n* = 71, 81%). PET data were corrected for scatter and randoms. Data were reconstructed using iterative OSEM reconstruction, with image quality parameters improving over the years.

Reconstructed PET images were re-evaluated visually by 1 specialist in nuclear medicine (LB), who was blinded to all other patient information. FDG PET uptake was scored visually at 7 vascular regions (thoracic and abdominal aorta, subclavian, axillary, carotid, iliac, and femoral arteries) as 0 (no FDG uptake), 1 (minimal but not negligible FDG uptake), 2 (clearly increased FDG uptake), or 3 (very marked FDG uptake). PET scans were considered positive in case of FDG uptake ≥grade 2 in any large vessel. If a patient had multiple PET scans during follow-up, the patient was considered to be PET positive during follow-up when one of the PET scans was positive.

### Statistical analysis

Categorical and continuous variables were expressed as count (percentage) and as median ± interquartile range (IQR) and range as appropriate. Given the expected bias and small patient number due to the fact that PET scans during follow-up were not part of the initial protocol, we only performed a descriptive analysis and did not perform statistical comparisons.

## Results

Eighty-eight repeat PET scans were performed in 52 out of 106 GCA patients, who were included in the original prospective cohort. Compared to patients without repeat PET scan during follow-up, GCA patients with a PET scan during follow-up included in this *post hoc* analysis had a longer symptom duration until diagnosis (7 vs 4 weeks), more frequently experienced relapse (92% vs 39%) and were treated longer (60 vs 24 months) with a higher cumulative glucocorticoid dose in the first 2 years (5.3 vs 4.1 g methylprednisolone) and more frequently with a glucocorticoid-sparing agent (46 vs 17%) (Table 1). There were no differences in age, sex, cardiovascular risk factors, symptoms, PET results, and temporal artery biopsy result at diagnosis and cardiovascular events during follow-up. Median time between PET scan at diagnosis and PET scans during follow-up was 28 months (IQR 17–56 months, range 2–109 months). Median daily methylprednisolone dose at time of repeat PET scan was 1 mg (IQR 0–4 mg, range 0–16 mg). Reasons for repeat PET imaging were described in Table 2. After interpreting all data (clinical signs and symptoms, acute phase response and PET results), 55 (63%) PET scans were done at the time of a relapse and 33 (38%) were done while the patient was in remission. At the time of relapse, 43 (78%) patients had PMR symptoms, 27 (49%) cranial symptoms, 35 (64%) constitutional symptoms and 3 (5%) patients had limb claudication. Among the patients with a positive PET scan at diagnosis (*n* = 37), 31 (84%) were persistently positive and 6 (16%) were negative during follow-up. Among the patients with a negative PET scan at diagnosis (*n* = 15), 12 (80%) remained negative and 3 (20%) were positive during follow-up.

**TABLE 1:** Comparison between patients with (included in this *post hoc* analysis) and without (excluded) repeat PET scan during follow-up.

Characteristics	Total (*n* = 106)	Included in *post hoc* analysis (*n* = 52)	Excluded from *post hoc* analysis (*n* = 54)
Age at inclusion, years, mean (SD)	70 (8)	70 (8)	70 (8)
Sex, no. of females, *n* (%)	70 (66%)	33 (63%)	37 (69%)
Symptom duration until diagnosis, week, median (IQR)	6 (3–14)^11^	7 (4–17)^7^	4 (2–9)^4^
**Cardiovascular risk factors/events at diagnosis**			
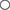 Active or past tobacco use, *n* (%)	48 (48%)^7^	26 (51%)^1^	22 (46%)^6^
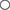 Antihypertensive medication, *n* (%)	51 (48%)	24 (46%)	27 (50%)
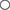 Statin use, *n* (%)	33 (31%)	12 (23%)	21 (39%)
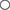 Diabetes mellitus, *n* (%)	5 (5%)	1 (2%)	4 (7%)
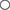 History of stroke, *n* (%)	5 (5%)	2 (4%)	3 (6%)
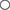 History of myocardial infarction or angina, *n* (%)	2 (2%)	0 (0%)	2 (4%)
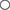 History of peripheral vascular, disease, *n* (%)	2 (2%)	1 (2%)	1 (2%)
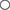 Betablocker at diagnosis, *n* (%)	39 (37%)	16 (31%)	23 (43%)
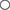 Betablocker during follow-up, *n* (%)	54 (51%)	25 (48%)	29 (54%)
AORTA score, median (IQR)	3.09 (2.97–3.22)	3.05 (2.94–3.21)	3.10 (2.98–3.22)
**Symptoms at diagnosis**			
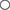 Cranial symptoms, *n* (%)	80 (75%)	39 (75%)	41 (76%)
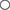 PMR, n (%)	39 (37%)	19 (37%)	20 (37%)
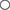 Constitutional symptoms, *n* (%)	93 (88%)	45 (87%)	48 (89%)
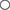 Limb claudication, *n* (%)	4 (4%)	3 (6%)	1 (2%)
**Laboratory tests at diagnosis**			
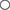 ESR, mm/h, median (IQR)	68 (48–105)^16^	93 (57–110)^3^	57 (45–70)^13^
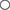 CRP, mg/l, median (IQR)	89 (48–135)	105 (46–157)	81 (48–129)
Positive temporal artery biopsy at diagnosis, *n* (%)	54 (64%)^22^	25 (60%)^10^	29 (69%)^12^
**PET results at diagnosis**			
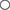 Positive PET, *n* (%)	75 (71%)	37 (71%)	38 (70%)
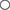 Positive PET in thoracic aorta, *n* (%)	61 (58%)	32 (62%)	29 (54%)
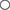 TVS, median (IQR)	7 (1–14)	8 (1–16)	6 (2–14)
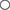 Number of vessels with FDG uptake ≥grade 2, median (IQR)	3 (0–5)	3 (0–5)	2 (0–5)
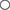 Intensity of FDG uptake in affected vessels, median (IQR)°	2.8 (0.0–3.0)	2.8 (0.0–3.0)	2.7 (0.0–3.0)
**Treatment**			
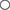 Duration of GC treatment, months, median (IQR)	33 (21–64)^3^	60 (33–96)^2^	24 (17–34)
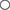 Cumulative GC dose in first 2 years after diagnosis, g methylprednisolone, mean (SD)	4.5 (3.7–5.4)^6^	5.3 (3.9–6.4)^4^	4.1 (3.6–4.8)^2^
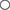 Use of glucocorticoid-sparing agents during follow-up, *n* (%)	32 (31%)^2^	23 (46%)^2^	9 (17%)
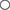 Duration of follow-up, months, median (IQR)	78 (40–110)	94 (64–120)	55 (29–95)
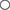 Relapse, *n* (%)	69 (65%)	48 (92%)	21 (39%)
**Cardiovascular event during follow-up**			
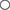 Aortic dissection, *n* (%)	0 (0)	0 (0)	0 (0)
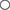 Vascular stenosis, *n* (%)	14 (13%)	10 (19%)	4 (7%)
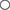 Vascular surgery, *n* (%)	7 (7%)	6 (12%)	1 (2%)
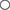 Myocardial infarction, *n* (%)	3 (3%)	2 (4%)	1 (2%)
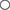 Stroke, *n* (%)	11 (10%)	6 (12%)	5 (9%)
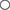 AION, *n* (%)	2 (2%)	1 (2%)	1 (2%)
Mortality, *n* (%)	15 (14%)	7 (13%)	8 (15%)

AION, anterior ischemic optic neuropathy; CRP, C-reactive protein; ESR, erythrocyte sedimentation rate; FDG, fluorodeoxyglucose; GC, glucocorticoids; IQR, interquartile range; no., number; PET, positron emission tomography; PMR, polymyalgia rheumatica; SD, standard deviation; TVS, total vascular score. Number of missing values are reported in superscript. °Calculated as total vascular score divided by the number of vessels with FDG uptake ≥grade 2.

**TABLE 2:** Reasons for repeat PET imaging stratified by disease activity.

*N* (%)	Total (*n* = 88)	Relapse (*n* = 55)	Remission (*n* = 33)
Suspected relapse	76 (86%)	55 (100%)	21 (64%)
Other disorders	5 (6%)	0 (0%)	5 (15%)
PET/CT scan performed instead of standalone CT scan	5 (6%)	0 (0%)	5 (15%)
Simultaneous participation to another study protocol	2 (%)	0 (0%)	2 (6%)

**FIGURE 1:**
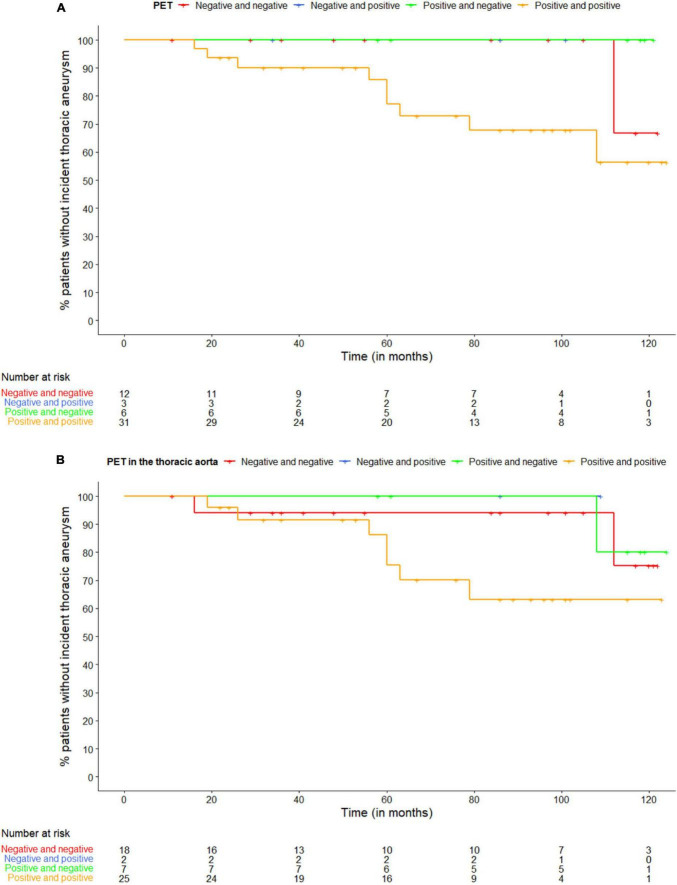
Kaplan-Meier curve of the proportion of patients with an incident thoracic aneurysm stratified according to panel **(A)** PET positivity in any large vessel and **(B)** PET positivity in the thoracic aorta. The first PET scan is at diagnosis, the second during follow-up.

Four out of 52 (8%) patients had a thoracic aortic aneurysm on the first CT scan, who all had both a positive PET scan at diagnosis and during follow-up. Ten (19%) out of 52 patients developed a thoracic aortic aneurysm during follow-up at a median time since diagnosis of 60 months (IQR 34–75), of which 9 patients had both a positive PET scan at diagnosis and during follow-up and 1 patient twice had a negative PET scan. Of the 9 patients with an incident thoracic aortic aneurysm and both a positive PET scan at diagnosis and during follow-up, 7 patients had twice a positive PET scan in the thoracic aorta, one patient had a positive PET scan in the thoracic aorta at diagnosis, but not during follow-up and one patient had twice a negative PET scan in the thoracic aorta. Figure 1 shows the Kaplan-Meier curve of the proportion of patients with an incident thoracic aortic aneurysm stratified according to PET positivity in any large vessel (Figure 1A) and PET positivity in the thoracic aorta (Figure 1B).

**FIGURE 2:**
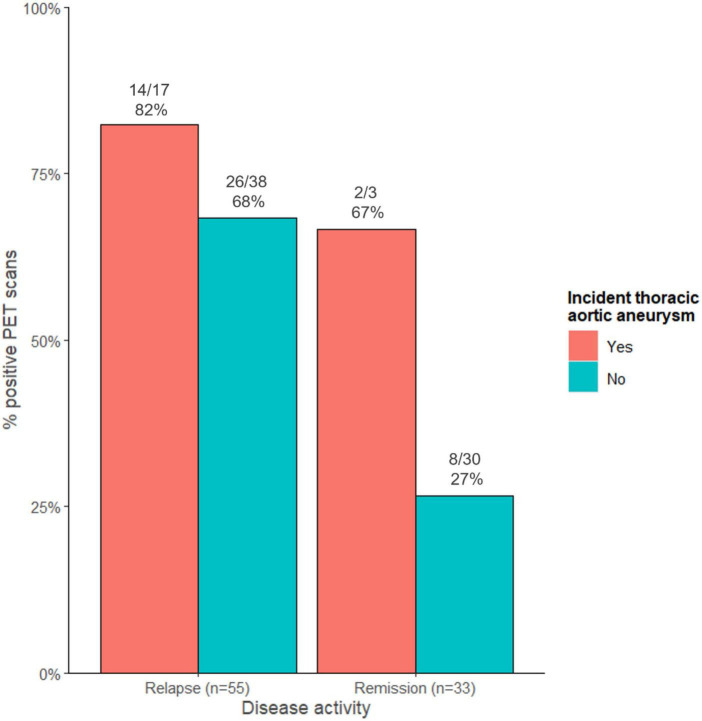
Proportion of positive PET scans stratified according to disease activity and to the presence of an incident thoracic aortic aneurysm.

Considering all 88 PET scans performed in these 52 patients, 14/17 (82%) PET scans performed during relapse were positive in patients with an incident thoracic aortic aneurysm compared to 26/38 (68%) PET scans in those without an incident thoracic aortic aneurysm (Figure 2). Of the PET scans performed while the patient was in remission, 2/3 (67%) PET scans were positive in patients with an incident thoracic aortic aneurysm compared to 8/30 (27%) PET scans in those without.

## Discussion

In our recently published prospective cohort study, we found that total vascular score at diagnosis was associated with a greater yearly increase in thoracic aortic diameters and volume (1). There was no association between the development of a thoracic aortic aneurysm and the treatment regimen or relapse rate. Hence, we concluded that the intensity and extent of the initial inflammation determines the risk for subsequent aortic dilatation.

However, in this *post hoc* analysis we found that nine out of ten incident thoracic aortic aneurysms occurred in GCA patients with both a positive PET scan at diagnosis and during follow-up. This suggests that ongoing aortic inflammation also may contribute to the development of thoracic aortic aneurysms in GCA. Since the majority of repeat PET scans in patients with an incident thoracic aortic aneurysm were performed at the time of a relapse, it is not possible to draw conclusions about FDG uptake in remission in these patients. However, our group earlier described the persistence of FDG uptake in a subset of treated asymptomatic GCA patients (3). In addition, recent reports of the group of Cornelia Weyand on the histopathological evaluation of repeat temporal biopsies (9) and aortic surgical specimens after aortic repair (6) showed ongoing vascular inflammation despite treatment and lack of clinical signs. This smoldering aortic inflammation may be caused by IFN-γ producing Th1 lymphocytes, which are more resistant to glucocorticoids in contrast to Th17 lymphocytes (10). Targeted therapies against Th1 cells may be necessary in addition to glucocorticoids to control chronic, smoldering vasculitis and to prevent the development of thoracic aortic aneurysms.

Four thoracic aortic aneurysms were already present on the first CT scan. These aneurysms may also be caused by smoldering vascular inflammation, which may have been already present prior to the development of symptomatic GCA.

The hypothesis that ongoing aortic inflammation may contribute to the development of thoracic aortic aneurysms in GCA is in contrast with the lack of association between occurrence of thoracic aortic aneurysms and treatment regimen or relapse rate. This may be explained by a difference in the number of relapses with large vessel vasculitis and relapses without large vessel vasculitis (e.g., isolated PMR or isolated cranial relapses). However, since there are no specific symptoms of large vessel vasculitis and since we did not routinely perform imaging at time of relapse, we could not prove this hypothesis.

Besides the limitations already mentioned in the original study (PET scans performed over a long time with increasing device quality, part of CT scans performed with contrast or on a PET/CT system, missing CT scans, single-center study and treatment at the discretion of the physician), this study has a major additional limitation. Repeat PET scans at predefined time points during follow-up were not included in the study protocol of the original prospective cohort study. The repeat PET scans during follow-up in this study were performed as driven by clinical practice, most frequently when a relapse was suspected. As a result, there is an important selection bias and the number of patients included is too small to draw solid conclusions. In addition, the data are very heterogeneous, both in terms of disease activity, disease duration since diagnosis and dose of glucocorticoids at the time of the repeat PET scan. Given these important limitations, we only performed a descriptive analysis and did not perform statistical comparisons.

In conclusion, nine out of ten patients with an incident thoracic aortic aneurysm, were PET positive at diagnosis and remained PET positive during follow-up. This finding suggests that in addition to the intensity and extent of the initial inflammation, ongoing aortic inflammation may contribute to the development of thoracic aortic aneurysms in GCA. However, this should be confirmed in a large prospective trial with repeat PET scans at predefined time points during follow-up.

## Data availability statement

The original contributions presented in this study are included in this article/supplementary material, further inquiries can be directed to the corresponding author.

## Ethics statement

The studies involving humans were approved by the Ethical Committee of the University Hospitals Leuven. The studies were conducted in accordance with the local legislation and institutional requirements. The ethics committee/institutional review board waived the requirement of written informed consent for participation from the participants or the participants’ legal guardians/next of kin because this was a purely retrospective study.

## Author contributions

DB: Writing – review and editing, Writing – original draft, Supervision, Methodology, Investigation, Formal Analysis, Data curation, Conceptualization. LM: Writing – review and editing, Writing – original draft, Visualization, Methodology, Investigation, Formal Analysis, Data curation. AB: Writing – review and editing, Writing – original draft, Visualization, Methodology, Formal Analysis, Data curation. SV: Writing – review and editing, Writing – original draft. WC: Writing – review and editing, Writing – original draft, Methodology, Investigation. LB: Writing – review and editing, Writing – original draft, Investigation, Formal Analysis. KL: Writing – review and editing, Writing – original draft.
